# Improved metrics for comparing structures of macromolecular assemblies determined by 3D electron-microscopy

**DOI:** 10.1016/j.jsb.2017.05.007

**Published:** 2017-07

**Authors:** Agnel Praveen Joseph, Ingvar Lagerstedt, Ardan Patwardhan, Maya Topf, Martyn Winn

**Affiliations:** a Institute of Structural and Molecular Biology, Department of Biological Sciences, Birkbeck College, University of London, Malet Street, London WC1E 7HX, United Kingdom; b Scientific Computing Department, Science and Technology Facilities Council, Research Complex at Harwell, Didcot OX11 0FA, United Kingdom; c European Molecular Biology Laboratory, European Bioinformatics Institute, Wellcome Genome Campus, Hinxton, Cambridge CB10 1SD, United Kingdom; d Computational Chemistry and Cheminformatics, Lilly UK, Windlesham GU20 6PH, United Kingdom

**Keywords:** 3D electron cryo-microscopy, Integrative modelling, Scoring functions, Macromolecular assemblies, Density fitting

## Abstract

Recent developments in 3-dimensional electron microcopy (3D-EM) techniques and a concomitant drive to look at complex molecular structures, have led to a rapid increase in the amount of volume data available for biomolecules. This creates a demand for better methods to analyse the data, including improved scores for comparison, classification and integration of data at different resolutions. To this end, we developed and evaluated a set of scoring functions that compare 3D-EM volumes. To test our scores we used a benchmark set of volume alignments derived from the Electron Microscopy Data Bank. We find that the performance of different scores vary with the map-type, resolution and the extent of overlap between volumes. Importantly, adding the overlap information to the local scoring functions can significantly improve their precision and accuracy in a range of resolutions. A combined score involving the local mutual information and overlap (LMI_OV) performs best overall, irrespective of the map category, resolution or the extent of overlap, and we recommend this score for general use. The local mutual information score itself is found to be more discriminatory than cross-correlation coefficient for intermediate-to-low resolution maps or when the map size and density distribution differ significantly. For comparing map surfaces, we implemented two filters to detect the surface points, including one based on the ‘extent of surface exposure’. We show that scores that compare surfaces are useful at low resolutions and for maps with evident surface features. All the scores discussed are implemented in TEMPy (http://tempy.ismb.lon.ac.uk/).

## 1 Introduction

A major leap in structure characterization of large bio-molecular machines and cellular components has been brought in by biophysical techniques like electron microscopy (EM) and tomography (ET) (Bai et al., 2013, Kuhlbrandt, 2014, Milne et al., 2013), which result in 3D volume representations of the structure. The Electron Microscopy Data Bank (EMDB) (http://emdb-empiar.org) currently holds over 4000 volume reconstructions from EM and ET, and the number of entries has doubled in the last four years due to increasing interest and development of better image reconstruction methods and direct electron detectors (Kuhlbrandt, 2014).

Rapid increase in the amount of EM/ET data necessitates ways to categorize and compare them. Comparison of 3D-EM reconstructions (volume alignment) is useful to categorize existing data and annotate new volume depositions. Conformational changes involved in specific biological systems can also be studied by comparing densities that represent different functional states.

The resolution of the 3D-EM data is often insufficient to provide atomic details of the macromolecular structure. Hence, atomic models of components are usually fitted into volumes to obtain an atomic representation of the structure (Villa and Lasker, 2014). Fitting atomic components into a target density is usually dealt with as a problem of volume alignment by first filtering the atomic model (probe) to the resolution of the target density before comparison (Chacon and Wriggers, 2002, Roseman, 2000, Rossmann, 2000, Topf and Sali, 2005, Volkmann and Hanein, 1999). Given an accurate placement derived from rigid-body alignment (rigid fitting), further refinement of the model can be applied locally by sampling conformations that improve the fit with the target density (flexible fitting) (Topf et al., 2008, Trabuco et al., 2008, Wang and Schroder, 2012).

The available approaches for fitting or volume alignment are either using a map density based 6D grid search or a coarse-grained representation of volumes to reduce the search space. Exhaustive 6D search of the density grid does not suffer from density approximations or coarse graining but is relatively slow. To reduce the computational cost, either the rotational search is accelerated using spherical harmonics transforms (Garzon et al., 2007) or a Fast Fourier Transform (FFT) is employed to rapidly scan the translations (Chacon and Wriggers, 2002, Roseman, 2000, Wriggers, 2012). Random (Goddard et al., 2007) or stochastic sampling (Topf and Sali, 2005) of the search space can also reduce computation time but is more effective when the probe and target volumes do not have a large difference in size. Cross-correlation coefficient (CCC) between the search and target map densities is typically the metric used in these methods to optimize the fit.

Methods relying on coarse-grained representation of volumes are faster but the accuracy largely depends on the efficiency of feature approximation. The molecular shape can be encoded with a set of feature points (Birmanns and Wriggers, 2007, Woetzel et al., 2011, Wriggers, 2012), even in the absence of interior density features. A least square fit starting from triplet points from feature sets (similar to geometric hashing) corresponding to probe and target, is performed to obtain the alignment. The sets of feature points are then compared using RMSD metric and the fit optimized using CCC score. Another approach based on vector quantization, represented density maps as alpha shapes that approximates the map geometry and topology (De-Alarcón et al., 2002). Volume densities are also described using 3D Zernike moments (Esquivel-Rodriguez and Kihara, 2012) and the Euclidean distance of the coefficients is computed to calculate the similarity of two volumes. Common features or substructures can be also derived using rotationally invariant local density gradient descriptors (Saha and Morais, 2012, Saha et al., 2010). The histograms of these local density gradient vectors are matched to compare the local density and the alignment is performed by matching graphs that representing the feature points.

GMfit (Kawabata, 2008) relies upon a representation of the map density in terms of a Gaussian Mixture Model (GMM), which is a linear combination of a certain number of 3D anisotropic Gaussian Distribution Functions (GDFs). A score based on the overlap of two Gaussian mixtures is optimized to obtain the alignment. The number of GDFs controls the description of the map; a larger number generates a more detailed density function. There are several ways to obtain initial configurations in the 6D search to align two GMMs. These include random sampling, segmentation-based or symmetric fitting, or by matching principal axes, followed by a local steepest descent optimization. The main computational cost is for the optimization of GDFs to generate the GMM while the comparison of Gaussian mixtures is usually carried out in seconds.

Apart from the alignment methodology, resolution, conformational differences and the extent of noise in the density maps also influence the efficiency of volume comparisons. A major factor that determines the selection of correct orientations in the search space is the accuracy of metric used to score the alignments (Farabella et al., 2015, Henderson et al., 2012, Schneidman-Duhovny et al., 2012, Volkmann and Hanein, 1999). It becomes necessary to evaluate and re-rank the proposed solutions using different scoring functions depending on the level of details in the volume reconstruction. Vasishtan and Topf (Vasishtan and Topf, 2011) presents an account of several scoring functions to evaluate the quality of alignment between two volumes, using either the density distribution of the volume or the shape of the surface of the density distribution contoured at a certain value or both. TEMPy is a Python toolkit for volume and model processing and assessment in which these scoring functions as well as additional ones are implemented (Farabella et al., 2015). Dugan and Altman assessed different scores for evaluating the match between a model and a surface envelope (Dugan and Altman, 2004). They proposed a metric favouring atom inclusion in the density while penalizing those lying outside the envelope. A similar score is used to evaluate fitted models associated with 3D-EM data depositions in EMDB (Lagerstedt et al., 2013).

In the context of the BioMedBridges project (Field et al., 2013), we have developed a pipeline for comparison of volumes to categorize and annotate existing volume data (PDBeShape; *to be published*). The precision and accuracy of scoring functions has been a major bottleneck in the assessment of solutions proposed by different volume comparison methods in this project. We therefore evaluated different scoring functions for their ability to distinguish correct volume alignments. We gathered a benchmark set of pairwise alignments of experimental 3D-EM reconstructions from the EMDB, using superposition of associated fitted coordinate models to provide a ground truth fit. We used GMfit (Kawabata, 2008) as the volume alignment method as it is relatively fast and a potentially useful method for volume database searches. For each pair of volumes, the set of alignments generated by GMfit were scored using different metrics and the metrics were then evaluated based on the similarity of the alignments with the reference fit. We tested potential improvements and normalization of the scoring functions discussed in (Vasishtan and Topf, 2011). We could characterise different scoring functions in terms of the class and resolution of the volumes involved, and the extent and nature of the overlap.

## 2 Methods

### 2.1 Dataset preparation

**Supplementary Fig. S1:**
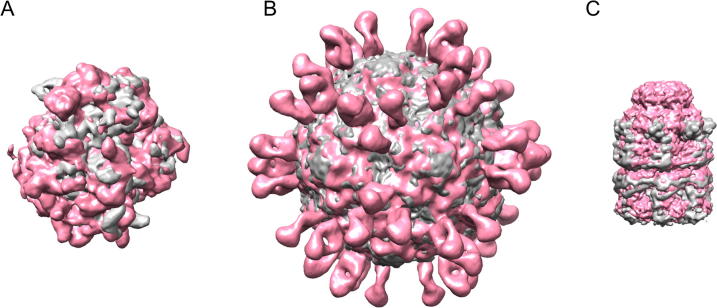
Examples of volume alignments from the dataset. (A) 13.8 Å map of Translation initiation complex from E.*coli* with 70S bound to IF1, IF3 and mRNA, EMD-1248 (grey) and 11.8 Å map of 80S ribosome from S.*cerevisiae* bound to eEF2, EMD-1067 (pink) (B) 5.5 Å map of Human poliovirus 1 135S particle, EMD-5710 (grey) and 10 Å map of Human enterovirus 71 in complex with neutralizing antibody E18, EMD-2397 (pink) (C) 8.5 Å map of GroEL from E.*coli*, EMD-2001 (grey) and 8.2 Å map of GroEL from E.*coli* bound to gp31 and ADP, EMD-1202 (pink). The map pairs shown were low-pass filtered to the lower of the resolutions of the two maps.

All 904 density maps (volumes) in the EMDB with corresponding fitted coordinate models in the PDB (as of April 2016) were considered. 50 maps each were chosen randomly from two major categories, ribosomes and viruses, and 30 maps for the categories of chaperones and other sample types; structure superposition of fitted atomic coordinates corresponding to maps in each category was carried out using MMalign (Mukherjee and Zhang, 2009) in order to define the ground truth alignments. For each alignment, MMalign calculates the TMscore which is a normalized score for evaluating the quality of superposition of atomic models, independent of the length of protein chains (Zhang and Skolnick, 2005, Zhang and Skolnick, 2004). Alignments with TMscore >0.4 (Xu and Zhang, 2010) were chosen and the transformation leading to superposition was used to transform the corresponding maps with respect to each other. However, even when the TMscore was good, the alignment of corresponding volumes could be non-optimal or incorrect due to fitting errors associated with one or both of the models and/or ambiguity in fitting at intermediate-low resolutions. We manually inspected the generated map-to-map alignments to remove those without correct matching orientations and selected a final reference set of 28 alignments (ribosome: 7, virus: 8, chaperones: 6 and various: 7) covering different resolutions and map types (Table 1). Examples of a reference alignment from each of the major categories are shown in Fig. S1. The 7 map-pairs not involving ribosomes, viruses or chaperones, includes two gamma secretase, one TRPV1 channel, one ryanodine receptor, one ATPase (Type-V) and two RNA polymerase pairs. For our category-based analysis of alignments, we included these 7 additional map pairs together with the 6 chaperone pairs in the category ‘others’.

**Table 1:** Dataset used for evaluating scoring functions. The EMDB IDs of the volumes aligned, their associated fitted PDBs (PDB1 and PDB2), sample category, their resolutions (resn1 and resn2) and the number of Gaussian functions used for each map (gmm1 and gmm2), are given. The fraction of overlapping region from the reference map alignment with respect to the size of each map, is given in the last column. GS: Gamma secretase, RPII: RNA polymerase II, RPIII: RNA polymerase III, RyR: Ryanodine receptor.

EMD1	EMD2	PDB1	PDB2	Category	Resn1	Resn2	gmm1	gmm2	Fractional overlap (m1, m2)
5247	5250	3izk	3izn	Other (chap)	4.9	6.4	32	16	0.61, 0.67
5247	5138	3izk	3j03	Other (chap)	4.9	4.8	122	104	0.67, 0.65
2001	1202	4aau	2cgt	Other (chap)	8.5	8.2	7	8	0.56, 0.53
2326	1202	3zq0	2cgt	Other (chap)	9.2	8.2	9	8	0.65, 0.73
2325	2326	3zpz	3zq0	Other (chap)	8.9	9.2	12	9	0.71, 0.85
5140	5248	3iyf	3izl	Other (chap)	8.0	6.2	11	12	0.31, 0.38
6455	5777	5an8	3j5r	Other (TRPV1)	3.8	4.2	33	45	0.84, 0.58
3240	2677	5fn5	3upc	Other (GS)	4.3	4.5	25	43	0.52, 0.86
2677	3061	4upc	5a63	Other (GS)	4.5	3.3	43	9	0.24, 0.87
2785	3218	4v1n	5flm	Other (RPlI)	7.8	3.4	23	41	0.60, 0.82
2786	3178	4v1o	5fj8	Other (RPIII)	9.7	3.9	23	23	0.36, 0.62
2752	2807	4uwe	3j8h	Other (RyR)	8.5	3.8	29	125	0.91, 0.46
8016	6284	5gar	3j9t	Other (ATPase)	6.4	6.9	11	10	0.44, 0.52
1302	1366	2o0f	1pn6	Ribosome	15.5	12.8	39	14	0.70, 0.69
1248	1067	1zo1	1s1h	Ribosome	13.8	11.7	10	16	0.72, 0.50
6456	5326	3jbn	3j0l	Ribosome	6.7	9.8	23	59	0.84, 0.36
2763	1895	3j81	4a2i	Ribosome	4.0	16.5	31	6	0.41, 0.74
1056	1895	1qzc	4a2i	Ribosome	9	16.5	19	6	0.20, 0.56
1345	5591	2p8z	3j38	Ribosome	8.9	6	15	110	0.77, 0.63
3049	2763	3jaq	3j81	Ribosome	6.0	4.0	27	31	0.66, 0.82
1182	1114	2c8i	1z7z	Virus	16	8	49	84	0.71, 0.90
5466	5122	3j23	3iyc	Virus	9.2	10	59	30	0.73, 0.73
5117	5268	3iya	3j05	Virus	22	7	72	74	0.24, 0.77
5710	2397	3j48	4c0u	Virus	5.5	10	60	72	0.48, 0.49
1058	5122	1upn	3iyc	Virus	18	10	126	30	0.37, 0.74
2436	1562	4c10	3epd	Virus	13	9	149	108	0.84, 0.43
5466	2397	3j23	4c0u	Virus	9.2	10	59	72	0.61, 0.52
6243	5888	3j9f	3jbc	Virus	9.0	6.5	153	82	0.54, 0.95

**Fig. 1:**
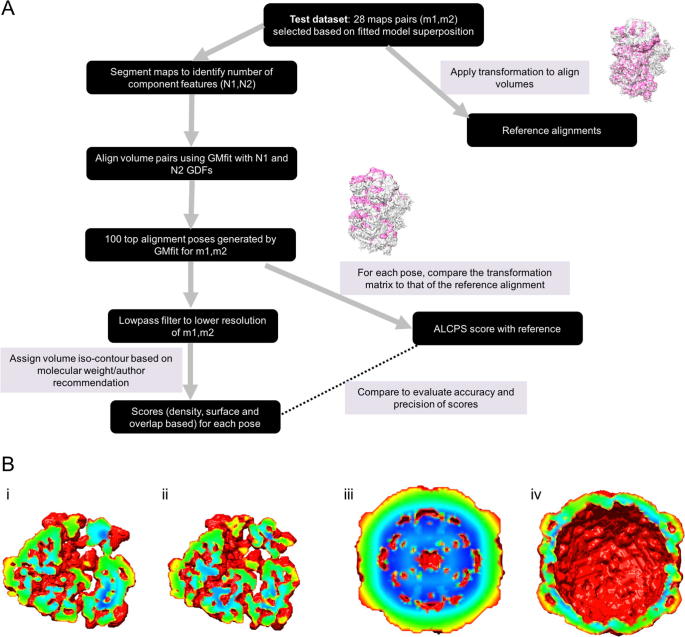
(A) Work flow of volume comparison with GMfit and assessment of alignment poses using different scoring functions. Reference alignment and one of the best fits generated by GMfit for the comparison of two partial yeast preinitiation complex maps (EMD-3049 and EMD-2763), are also added as examples. (B) Mean filter applied on binary mask of contoured volumes to identify surface exposed points. Applying a mean filter on a binary mask of contoured volume result in voxel values between 0 and 1.0, more exposed points close to 0 and the core voxels close to 1.0. A slice through such a filtered volume is shown with voxel values colored in the rainbow range with red indicating maximum exposure and blue, maximum burial. (B.i) 15.5 Å cryo-EM map of E-coli 70S ribosomal release complex bound to RF3 (EMD-1302). (B.ii) 12.8 Å map of EF-G bound E-coli 70S Release Complex in the presence of Puromycin and GTP (EMD-1366). (B.iii) 18 Å map of echovirus type 12 bound to decay accelerating factor (EMD-1058) and (B.iv) 10 Å map of human poliovirus 1 RNA-releasing intermediate (EMD-5122).

We next used GMfit (Kawabata, 2008) to generate 100 alignments for each map pair in the reference set (Fig.1A), in order to provide a distribution of scores in each case. Generally, the larger the number of Gaussians used to represent a volume, the more features can be abstracted and the better is the description of the density. The number of Gaussians used to approximate a map was inferred from the *number of segments* found by Segger (Pintilie et al., 2010). Segger (implemented in Chimera (Pettersen et al., 2004)) is widely used for segmenting volumes to identify component shapes in the volume density. An initial application of the watershed algorithm is followed by iterative scale-space filtering and grouping resulting in larger and fewer segments.

In our implementation, we terminated the grouping when the observed number of segments falls below an expected number. We calculated the lower limit for the approximate number of segments expected from the volume by estimating the theoretical protein volume corresponding to 100 amino acids (Harpaz et al., 1994) and scaling it by a factor of map resolution

(1) SV=(100∗110∗1.21)∗r

Here, *SV* is the effective volume of a segment in Å^3^, scaled by the resolution *r* (in Å) of the map. 110 Da was used as the average molecular weight of each amino acid and 1.21 Å^3^/Da is the factor obtained by considering an average partial specific volume of ∼0.73 cm^3^/g, for proteins. Dividing the total molecular volume by this scaled segment volume gives the number of segments expected, with the Segger procedure terminating at the next iteration at some number lower than this. The latter was then taken as the number of Gaussians to be used in GMfit. The theoretical volume is scaled by the map resolution *r* so that a lower resolution map is represented by fewer Gaussians compared to a higher resolution map. In practice, we also imposed a minimum number of segments of 3 and a maximum number of 240. The selected number of Gaussians for each volume in the test dataset is given in Table 1. To match volumes abstracted by different Gaussian mixtures, the random search protocol in GMfit was used, followed by a steepest descent local optimization (Kawabata, 2008).

As explained below, score calculations require assignment of an appropriate contour level for the volumes so that the density beyond this level can be considered as background noise. We determined contour density threshold based on the volume corresponding to the molecular weight of the macromolecule. For EMDB entries the molecular weights details provided by the authors may not be accurate or may not account for all components in the sample. Hence, we also considered the author-suggested contour level, and calculated the molecular volume from that. If the contour calculated from the estimated molecular weight falls below the background peak or above 5*sigma (where sigma is the standard deviation of density values, calculated with the background peak as mean), then the author-suggested contour value is used instead. If the author-suggested level also fails the sanity check, then 1.5*sigma above the background peak is used. Also, if the contour level based on the molecular weight (as submitted to EMDB) differed significantly from the contour suggested by the authors (>3 sigma), the latter was used. The selected contour levels were manually verified. Next, we low-pass filtered the maps to the lower resolution of the two, using TEMPy (Farabella et al., 2015) and the grid spacing was set to 1/4 of that resolution (van Zundert et al., 2017). The transformation proposed by GMfit for each fit was applied to the maps, followed by the calculation of different scores. The accuracies of different scoring functions (below) were then assessed using these proposed alignments with respect to the reference alignment. All the scores, band-pass filters and grid resampling functions are implemented in TEMPy (Farabella et al., 2015).

The following scores were selected based on their performance in previous tests on a simulated map dataset (Vasishtan and Topf, 2011) and the differences in the features they score, e.g. the voxel density values, binned densities, map surface features, surface density gradients, extent of overlap etc. Potential modifications and improvements to these scores (see below) were also tested in this analysis.

### 2.2 Density-based scores

The following scores consider the voxel density values for calculations. Local score calculations are carried out over all voxels that are within the contour of both maps, i.e. the overlap region.

The *global Cross Correlation* (CCC) was calculated as:

(2) $$\mathrm{CCC}=\frac{\sum (x-\overset{¯}{x})(y-\overset{¯}{y})}{\sqrt{\sum {\left(x-\overset{¯}{x}\right)}^{2}}*\sqrt{\sum {\left(y-\overset{¯}{y}\right)}^{2}}}$$

where x and y are density values in each voxel in the two volumes being compared and $\overset{¯}{x}$ and $\overset{¯}{y}$ are the respective mean densities. The *Local Cross Correlation* (SCCC) is calculated as:

(3) $$\mathrm{SCCC}=\frac{\sum (x-\overset{¯}{x})(y-\overset{¯}{y})}{\sqrt{\sum {\left(x-\overset{¯}{x}\right)}^{2}}*\sqrt{\sum {\left(y-\overset{¯}{y}\right)}^{2}}}$$

where the summation is limited to the set of voxels in the region of overlap. To make it less sensitive to the local differences in the shape of density distributions and the location of the mean (Joseph et al., 2016), another local score implemented in TEMPy, *Segment based Manders’ Overlap Coefficient*, was calculated as the product moment without deviation from mean (as also used in Chimera):

(4) $$\mathrm{SMOC}=\frac{\sum (\mathrm{xy})}{\sqrt{\sum {\left(x\right)}^{2}}*\sqrt{\sum {\left(y\right)}^{2}}}$$

The local nature of the score calculation is similar to the segment-based cross-correlation score (Farabella et al., 2015).

Another score that performed well in previous tests on simulated maps, and works on a coarser representation of density (in terms of density bins), is the mutual information (MI) score (Shatsky et al., 2009, Vasishtan and Topf, 2011). It quantifies the extent of register between the density bins from the two maps.

The *Local Mutual Information* (LMI) was calculated in a way similar to that described in (Farabella et al., 2015), for the region of overlap. The volume density was divided into a certain number of bins calculated using Sturges rule (Sturges, 1926)) as

(5) $$k=[1+\log_{2}n]$$

where *k* is the number of bins and *n* is the number of voxels in the overlapping region. For this test dataset, the number of bins *k* usually stayed close to 20, corresponding to a typical overlap region of 80^3^ voxels. The marginal entropies *H_X_* and *H_Y_* for the two aligned maps were calculated as:

(6) $$H_{X}=-\overset{k_{x}}{\underset{x=1}{\sum }}p_{x}*\log_{2}(p_{x})$$

(7) $$H_{Y}=-\overset{k_{y}}{\underset{y=1}{\sum }}p_{y}*\log_{2}(p_{y})$$

where p_x_ and p_y_ are the probabilities of occurrence of the corresponding bins (x and y) in the sample and k_x_ and k_y_ are the number of bins into which the volume densities were divided. The joint entropy of aligned bins from the two volumes was calculated as:

(8) $$H_{\mathrm{XY}}=-\overset{k_{x}}{\underset{x=1}{\sum }}\overset{k_{y}}{\underset{y=1}{\sum }}p_{\mathrm{xy}}*\log_{2}(p_{\mathrm{xy}})$$

where p_xy_ is the probability of finding the pair of bins x, y in the aligned set of bins from the two volumes.

The Mutual Information score was then calculated as:

(9) $$\mathrm{MI}=H_{X}+H_{Y}-H_{\mathrm{XY}}$$

It captures the statistical relationship between the two binned densities based on their joint entropy. The joint entropy is minimized when there is a one-to-one mapping between the bins.

A decrease in overlap between volumes reduces the statistical power of estimated probabilities. *Normalised Mutual Information* (NMI) (Studholme et al., 1999) was designed to make the global Mutual Information score less variant to changes in the extent of overlap:

(10) $$\mathrm{NMI}=(H_{X}+H_{Y})/H_{\mathrm{XY}}$$

### 2.3 Surface-based scores

Using a given contour level, the surface points of a volume can be picked in different ways. Various surface definitions were tested:

a) *Based on a density threshold (T)*. All voxel points whose density lie in a given range are selected as surface points (Vasishtan and Topf, 2011). In this study, we used contour level ±10% sigma as the density range.
b) *All points on the contour surface (A)*. On a contoured volume filled with zeros outside the surface, the set of voxels with at least one zero in the immediate neighbourhood are considered as surface points. The face, edge and corner contacts were considered while searching the neighbourhood.
c) *Mean filter for identifying extent of exposure (M)*. Based on the chosen contour level, a binary mask is generated from the density map with ones inside and zeros outside the contour. Every voxel value within the contour is then replaced with the mean of mask values over three orthogonal windows of length 21 voxels, lying along the map axes and centred on the point of interest. We chose this window size because we deal with large volumes (size > 100^3^ voxels) and a larger window enables calculation of the extent of exposure/burial based on a larger neighbourhood. As a result, highly exposed voxels surrounded by more exterior points get a low value compared to those on grooves or in pockets (Fig.1B). All voxels with values less than 0.3 were then selected as surface points. This provides a simple way to extract the surface and compare aligned volumes based on the extent of surface exposure.

The following scores rely on surface definitions to calculate similarity.

The *Chamfer Distance* is used for pattern matching in video tracking, and is calculated as the average Euclidean distance between nearest surface points taken from two volumes (Chen et al., 2007, Vasishtan and Topf, 2011). We calculated the Chamfer Distance for surface points identified using the three methods described above, giving the scores CDT, CDA and CDM.

For atomic structures, the Global Distance Test (GDT) score is computed as a weighted percentage of Cα atom pairs in a given distance range (Zemla, 2003, Zemla et al., 2007). GDT has been widely accepted as a measure for the quality of superposition of two coordinate sets representing protein structures, and this score is used to evaluate computational models in the CASP (Critical Assessment of protein Structure Prediction) experiments (Read and Chavali, 2007). Here, by analogy to the GDT, we calculate an additional score based on the Chamfer distance as a weighted mean of the fraction of surface point pairs within a certain distance. For a set of equi-spaced distance limits D(*i*) (a maximum distance divided into k equal bins), the CD_GDT_ score is given by

(11) $${\mathrm{CD}}_{\mathrm{GDT}}=\frac{\overset{k}{\underset{i=1}{\sum }}[(k-i+1)*P_{i}]}{k^{*}(k+1)/2}$$

where P_i_ is the fraction of nearest point pairs within the distance limit D(*i*).

A maximum distance threshold of 30 Å was used, and the nearest neighbour distances were placed into k = 30 bins of width 1.0 Å. The weight for the *i*th bin is $\frac{\left(k-i+1\right)}{k^{*}(k+1)/2}$ such that the weight falls linearly with increasing distance, dropping to zero for nearest neighbour distances greater than the maximum distance threshold. We calculated CD_GDT_ using all three surface definitions described above: CDT_GDT_, CDM_GDT_ and CDA_GDT_, respectively.

The *Normal Vector* score was calculated as the average angle between the normal vectors at aligned surface points (Ceulemans and Russell, 2004, Vasishtan and Topf, 2011), normalized as:

(12) $$\mathrm{NV}=\frac{1}{\left(n^{*}\pi \right)}\overset{n}{\underset{i=1}{\sum }}\frac{\overset{\to }{N_{i}^{x}}\times \overset{\to }{N_{i}^{y}}}{\left(|\overset{\to }{N_{i}^{x}}||\overset{\to }{N_{i}^{y}}|\right)}$$

where *n* is the number of surface points of the target volume, $\overset{\to }{N_{i}^{x}}$ and $\overset{\to }{N_{i}^{y}}$ are normal vectors of density gradients calculated at these points *i* for the two maps *x* and *y*. The score varies from 0 for perfectly aligned and parallel surfaces, up to the worst score of 1.

We calculated the Normal Vector score using all three surface definitions: NVT, NVM and NVA respectively.

### 2.4 Overlap-based scores

The final score relies on quantifying the overlapping regions between the two maps irrespective of the density values inside the contour. The *Overlap score* (OVR) is calculated as the fraction of overlapping voxels within the iso-contour threshold with respect to the smaller of the two volumes.

### 2.5 Measures for evaluating scores

To compare different scores, we used them to evaluate each of the 100 fits generated by GMfit for a pair of maps. The distance of each of these 100 fits from the reference alignment was measured using the Arc Length corresponding to the Component Placement Score (CPS) (Pandurangan et al., 2014, Zhang et al., 2010): ALCPS ≡ 2π*r*θ/360, where *r* is the translation vector and θ is the angle corresponding to the difference in transformations between the reference and current fit. For symmetric maps, the symmetry operations were considered while calculating this metric. The logarithm (log_10_) of ALCPS was used for the following analyses and plots.

To determine the ability of a score to distinguish alignment poses that are close to the reference alignment from those farther from it, we measured the ALCPS values for each alignment from GMfit. We considered a certain ALCPS threshold: alignments were considered as “correct” if the associated ALCPS is better than the threshold. For each score being tested, alignments with a score greater than a score threshold are considered positives and the rest as negatives. The true positive rate (TPR) is the fraction of correct alignments that are recovered as positives. Similarly, the false positive rate (FPR) is the fraction of incorrect alignments that are reported as positives. The true and false positive rates are measured as a function of the score threshold, for each score being tested. Receiver Operating Characteristic (ROC) curves which plot the TPR against the FPR as the score threshold is varied, were generated for each of the scores and for each map pair in the test dataset. The mean Area Under Curve (AUC) of all ROC curves in the test dataset was calculated, with larger values indicating a clean separation of true positives from false positives. AUC values reflect here the ability of a score to discriminate between correct and incorrect alignments. However, when the number of incorrect fits is significantly higher than the number of correct fits (or *vice versa*), the differences in the TPR between two scores will appear more dominant compared to that of the FPRs (or *vice versa*) (Davis and Goadrich, 2006). The ROC curves and the AUC values can be biased in such cases. Hence, we also calculated the fraction of true positives (correct fits) among the reported positives, which is the precision of each score.

We calculated accuracy and precision at different score thresholds and report the precision at the score threshold associated with the maximum accuracy (threshold at which a better separation of correct (true) and incorrect (false) fits is observed). For a given score and at a selected score threshold, the accuracy is calculated as:

(13) (TP+TN)/(TP+TN+FP+FN)

while the precision is calculated as:

(14) TP/(TP+FP)

where TP, FP, TN and FN refers to the number of true positives, false positives, true negatives and false negatives. Higher AUC and precision reflect fewer false positives and false negatives.

The above statistical measures were based on an assumed ALCPS threshold distinguishing correct from incorrect fits, which provides the ground truth for assessing individual scores. We have also varied the ALCPS threshold in order to make the criterion for a correct alignment more or less strict.

## 3 Results and discussion

**Supplementary Fig. S2:**
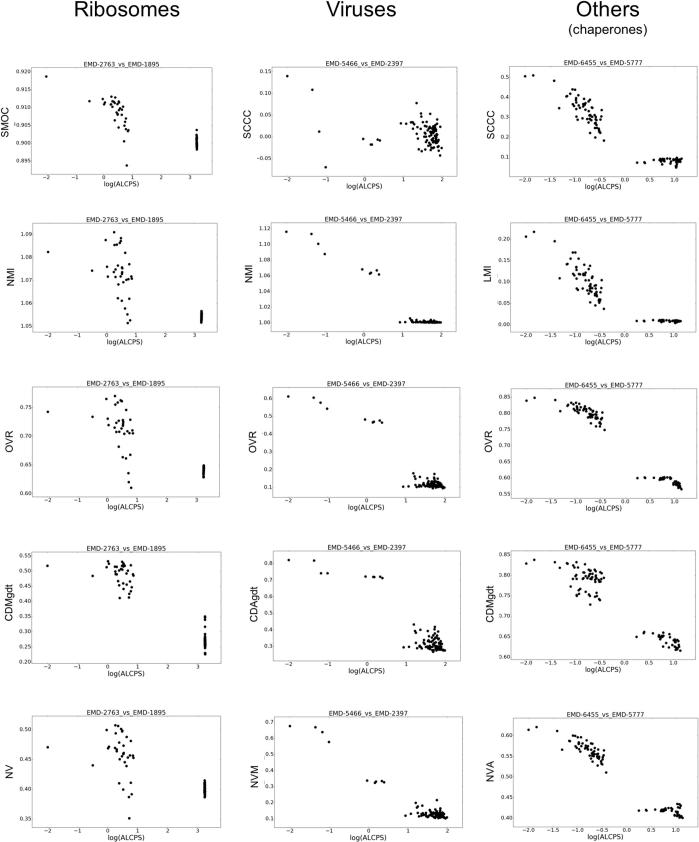
Examples of scores vs ALCPS (log_10_ scale), from the three categories: (A) ribosomes: EMD-2763 vs EMD-1895. Fits with ‘correct’ orientations are below log_10_(ALCPS) 1.0. (B) viruses: EMD-5466 vs EMD-2397. Fits with ‘correct’ orientations are below log_10_(ALCPS) -0.5. (C) chaperones: EMD-5247 vs EMD-5250. ‘correct’ fits are below log_10_(ALCPS) -0.5. Each point represents one of the 100 fits generated by GMfit, except for the dot in the top left-hand corner which indicates the reference fit (The reference alignment is assigned a minimum log_10_(ALCPS) value of −2.0 for the purpose of plotting). ALCPS measures the distance of a fit from the reference alignment, with lower values indicating better fits. See the Methods for an explanation of the different scores shown here. In each category, the scores are selected to highlight differences in their ranking of the fits for these specific examples from each category.

We first designed an automated way to determine the number of Gaussian density functions (GDFs) to be used in GMfit based on the number of segments that can be identified from the volume density (see Methods). This is required as GMfit is sensitive to the number of GDFs included in the Gaussian mixture model (GMM) approximating the volume density (Kawabata, 2008). With the resulting GMMs, we used GMfit to generate 100 volume alignments for each map pair in the test dataset. The best alignment (closest to reference as judged by the ALCPS value) was in the top 20 fits from GMfit, for 25 out of 28 map pairs. In one case, the best alignment is not ranked highly by GMfit, suggesting that the GMMs used might not be optimal. In the other three cases, GMfit failed to generate any optimal or near optimal alignment in the top 100 solutions. Only the reference alignment obtained from superposition of fitted models is considered reliable in these cases. For the majority of map pairs we obtain a few fits or a cluster of fits from GMfit which are close to the reference (low ALCPS), separated from a cluster of alignments representing bad fits (high ALCPS) (e.g. see Fig. S2). For viral maps and the category ‘others’, symmetry related fits were often found among the correct set of fits.

**Fig. 2:**
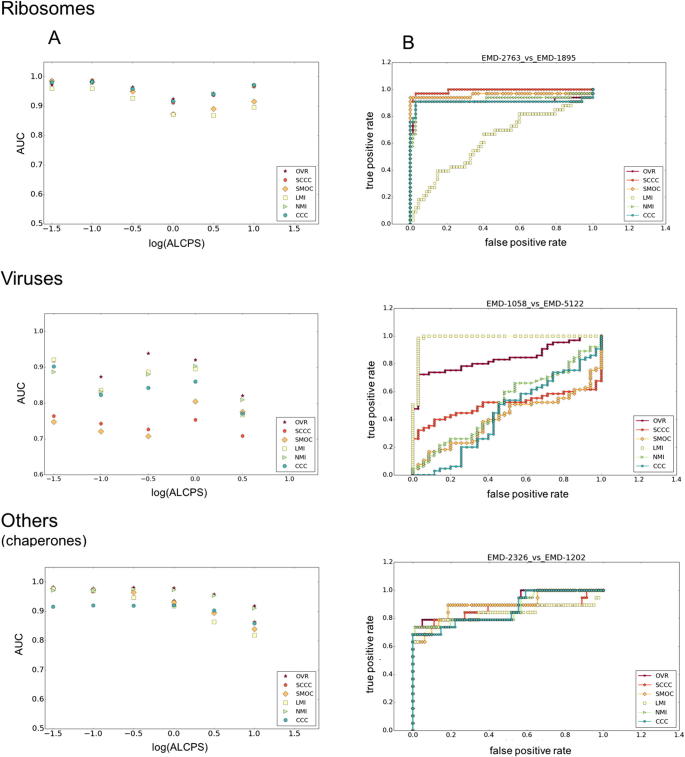
Panel A gives the average AUC of selected scores for the ribosomes, viruses and the category ‘others’ (involving chaperone structures), as a function of the ALCPS threshold used to identify correct alignments. The average AUC was calculated based on ROC curves from the test dataset, with larger values implying better discrimination between correct and incorrect fits. Larger values of log_10_(ALCPS) reflect a relaxed criterion for a correct fit. For most of the viral map alignments (except 3 cases: EMD-5466 vs EMD-2397, EMD-1182 vs EMD-1114 and EMD-5466 vs EMD-5122), none of the fits among the 100 GMfit solutions (including incorrect fits), had ALCPS score > 1.0. Hence the plots for viral maps are restricted to a maximum ALCPS threshold of 0.5. Panel B shows examples of ROC curves taken from the three structural categories, calculated using selected threshold for log_10_(ALCPS) for each category (ribosomes: 0.82, viruses: −0.5, ‘others’: −0.4). OVR: Overlap score, LMI: Local mutual information, NMI: Normalized mutual information, SCCC: Local cross correlation, SMOC: Local cross correlation about zero.

**Supplementary Fig. S3:**
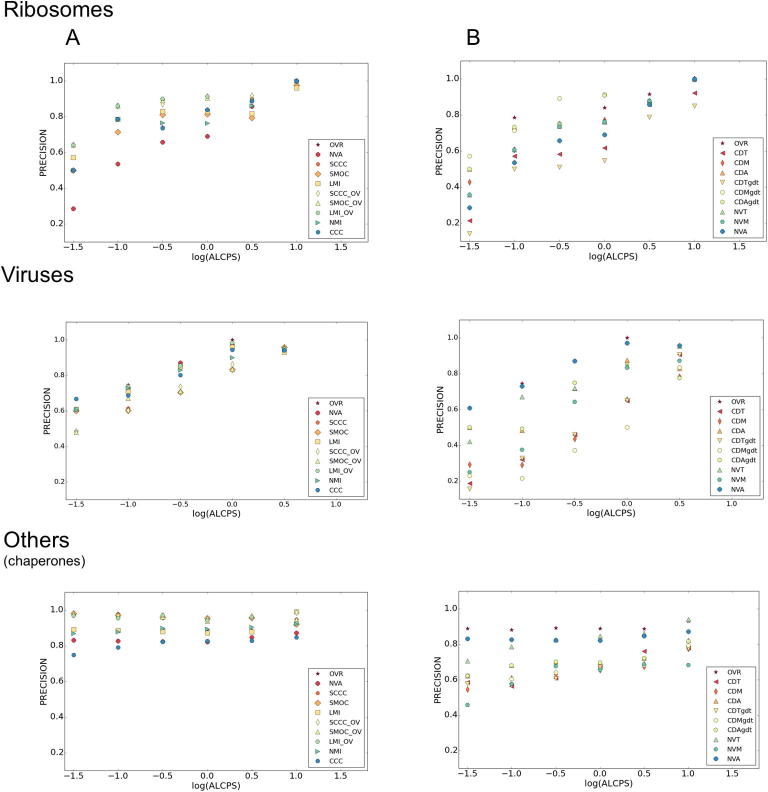
Precision of scores vs distance from reference. (A) and (B) gives the precision of density and surface-based scores for ribosomes, viral and ‘others’ (involving chaperones) alignments respectively. The log_10_ of ALCPS metric is used to show the distance of each alignment from reference. The average precision was calculated from values at different ALCPS thresholds. For most of the viral map alignments, none of the fits among the 100 GMfit solutions (including incorrect fits), had ALCPS score > 1.0. Hence the plots for viral maps are restricted to a maximum ALCPS threshold of 0.5.

Using the distribution of fits from GMfit and the resultant ROC curves, we evaluated the ability of different scores to discriminate between true and false positives. We computed Area Under the Curve (AUC) of each score at different levels of required similarity to the reference alignment, as set by the ALCPS threshold (Fig.2A). As the fit moves farther from the reference, the log_10_(ALCPS) values go from negative to positive. Similar orientations were usually observed for poses with log_10_(ALCPS) up to around 0.0 ± 0.5 (Fig. S2), i.e. small rotations and translations from the reference. For example, log_10_(ALCPS) ∼ 0.0 roughly corresponds to a shift of 6 Å with a rotation of 10°, or a shift of 10 Å with a rotation of 6°. There is some variation in the performance of the scores with the choice of ALCPS threshold, depending also on the structural category. Hence we considered log_10_(ALCPS) thresholds specific for each category such that the threshold distinguishes the cluster of correct orientations for most of the fits in that category. We used threshold values of 0.82, −0.50 and −0.40 for ribosomes, viruses and the category ‘others’, involving chaperones, respectively. The AUC shows different trends in each category, as the threshold is loosened (Fig.2A). For ribosomes and viral maps, as the fits with correct orientation moved away from the reference fit, the AUC dropped initially before raising (Fig.2A). This is largely due to the fact that at intermediate to low resolutions, an ensemble of similar orientations typically has comparable scores (Farabella et al., 2015, Goulet et al., 2014, Lukoyanova et al., 2015). On the other hand, the precision of scores generally improves for all structural classes as the criterion for a good fit is weakened (Fig. S3). Nevertheless, in general our conclusions about the relative merits of different scores are independent of the ALCPS threshold used.

### 3.1 Differences in density distribution and composition

**Fig. 3:**
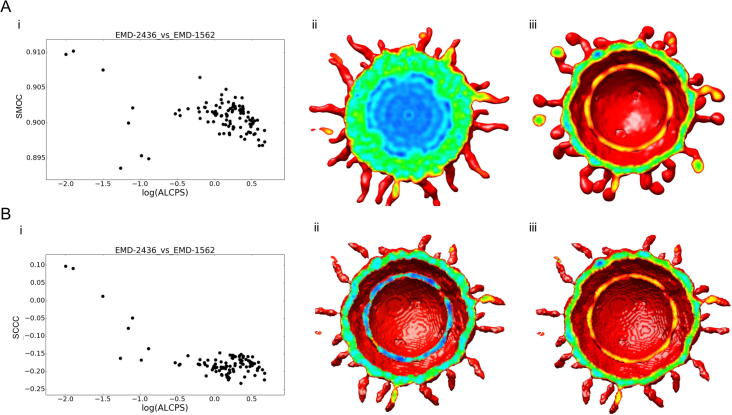
Alignment of 13 Å map of human enterovirus 71 in complex with antibody E19 (EMD-2436) and 9 Å map of human poliovirus 3 (EMD-1562). (A.i) shows the SMOC scores of top 100 GMfit alignments with respect to deviation from reference alignment in terms of ALCPS scores, while (A.ii) and (A.iii) shows the cross section of the two viral maps EMD-2436 and EMD-1562 colored in rainbow based on the density values, red indicating low density and blue, high density. (B.i) SCCC scores of top 100 GMfit alignments with respect to deviation from reference alignment in terms of ALCPS scores. (B.ii) and (B.iii) region of overlap of the reference alignment of the maps, with the mean difference of density values colored in rainbow, red highlighting the minimum (negative) and blue, maximum (positive).

For viral maps, the global NMI score has better AUC values and precision than the global CCC score. LMI was also better than CCC/SCCC/SMOC scores for viral map alignments. The comparison of viral maps often involves significant compositional differences due to DNA/RNA packaging (e.g. the empty human enterovirus 71 (EMD-2436) vs the RNA-containing human poliovirus 3 (EMD-1562)) and/or decorations of the viral envelope (e.g. the immature Dengue virus (EMD-5117) vs Dengue virus serotype 1 complexed with HMAb (EMD-5268)) (Figs.2B and 3). For these and a few other viral map comparisons in the test dataset only a part of one map or parts of both maps were comparable reflecting partial overlap (Table 1). Hence a local score such as LMI is expected to be better in such cases, due to the fact that the non-overlapping regions had significant differences.

For the alignment between human enterovirus 71 (EMD-2436) (Plevka et al., 2014) and human poliovirus 3 (EMD-1562) (Zhang et al., 2008), two maps for which the density distributions are not comparable, the AUC of SCCC was significantly higher than that of SMOC (Figs.2B and 3). Poliovirus is structurally similar to other enteroviruses, with a non-enveloped icosahedral protein coat encapsulating an RNA genome. The enteroviral volume core is empty compared to the high-density core of the polioviral map (Fig.3A.ii & iii) and the capsids have similar diameters. The surface protrusions involving relatively lower density values are important in differentiating the correct alignment from the rest. Mean difference in the region of overlap helped to match these low-density surface features (both negative after mean difference) with a raise in score (Fig.3B.ii & iii). Equivalent minimal densities however have a relatively lower contribution to SMOC, which involves product of absolute densities.

### 3.2 Differences in surfaces

**Fig. 4:**
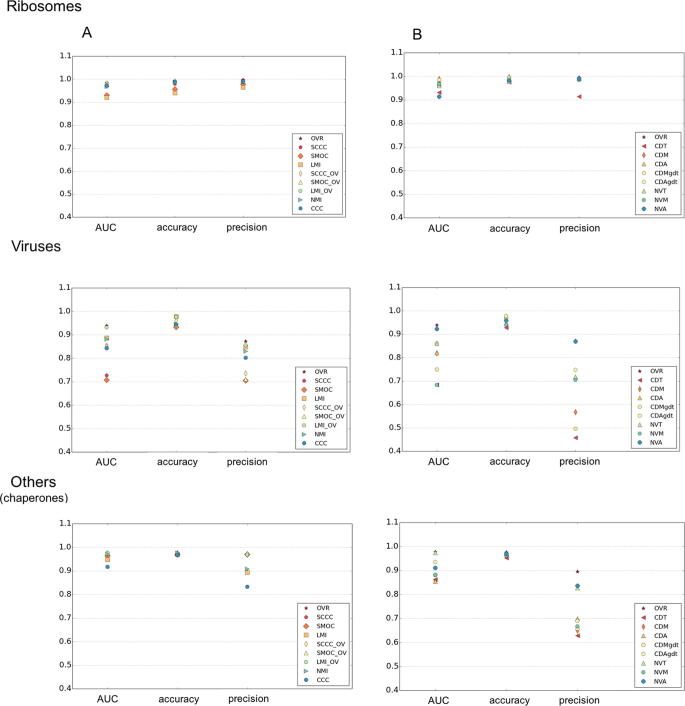
Performance of density and surface based scores in each category. For ribosomal, viral and the ‘others’ (including chaperones) categories, the AUC, accuracy and precision of density (left) and surface based (right) scores are plotted. The AUC, accuracy and precision values are calculated at log_10_(ALCPS) thresholds selected for each category (ribosomes: 0.82, viruses: −0.5, ‘others’: −0.4). OVR: Overlap score, LMI: Local mutual information, NMI: Normalized mutual information, SCCC: Local cross correlation, SMOC: Local cross correlation about zero. The combined scores with OVR are indicated with the ‘_OV’ tag. CDT: Surface distance score on points selected based on a density threshold range. CDM: Surface distance score on points selected using mean filter (to identify more exposed regions), CDA: Surface distance score on all points at an iso-contour level, CDMgdt & CDAgdt scores are normalized variants of CDM & CDA (see Methods), NVM: Normal vector score on surface points identified by mean filter on binary mask, NVA: Normal vector score on all points at an iso-contour level.

The two new filters applied for surface envelope detection (surface definition M and A, see *Methods*) significantly improved AUC of surface-based scores, compared to those used previously which were based on a contour threshold range (surface definition T) (Farabella et al., 2015, Vasishtan and Topf, 2011) (Table 2). The selection of surface points in a range of contour thresholds (definition T) is affected by the relative spatial variation of density levels at the surface. Considering all points on the iso-contour surface touching at least one exterior voxel (surface definition A) generally resulted in a better performance than the other surface envelope definitions (Fig. 4, Table 2).

**Table 2:** For the three map categories, the average AUC value, accuracy and precision of each score, are given. These were calculated at log_10_(ALCPS) thresholds selected for each category (ribosomes: 0.82, viruses: −0.5, others: −0.4). The scores which are better discriminatory and/or have higher precision, are in bold. OVR: Overlap score, LMI: Local mutual information, NMI: Normalized mutual information, SCCC: Local cross correlation, SMOC: Local cross correlation about zero. The combined scores with OVR are indicated with the ‘+OV’ tag. CDT: Surface distance score on points selected based on a density threshold range. CDM: Surface distance score on points selected using mean filter (to identify more exposed regions), CDA: Surface distance score on all points at an iso-contour level, CDTgdt, CDMgdt & CDAgdt scores are normalized variants of CDT, CDM & CDA (see Methods), NVT: Normal vector score on surface points selected from a density threshold range, NVM: Normal vector score on surface points identified by mean filter on binary mask, NVA: Normal vector score on all points at an iso-contour level.

Scores	Ribosome			Virus			Others		
	AUC	Accuracy	Precision	AUC	Accuracy	Precision	AUC	Accuracy	Precision
OVR	0.974	**0.990**	**0.991**	**0.939**	**0.971**	**0.872**	**0.978**	**0.977**	0.895
SMOC	0.931	0.957	0.979	0.707	0.933	0.705	0.964	0.969	**0.970**
SCCC	**0.983**	0.980	**0.997**	0.727	0.936	0.705	0.946	0.969	**0.969**
CCC	0.973	**0.990**	**0.991**	0.843	0.945	0.802	0.917	0.969	0.834
LMI	0.901	0.923	0.941	0.887	0.979	0.848	0.948	0.970	0.894
NMI	0.969	**0.989**	**0.991**	0.881	0.946	0.830	0.972	**0.976**	0.909
CDT	0.931	0.976	0.914	0.685	0.929	0.458	0.875	0.952	0.629
CDM	0.954	**0.991**	0.920	0.801	0.970	0.436	0.856	0.962	0.649
CDA	0.967	**0.990**	**0.991**	0.821	**0.976**	0.717	0.854	0.970	0.700
NVT	0.960	0.979	0.990	0.861	0.948	0.719	0.974	0.973	0.827
NVM	0.978	0.984	0.984	0.701	0.939	0.643	0.882	0.962	0.667
NVA	0.914	0.983	**0.990**	0.922	0.959	**0.870**	0.911	0.970	0.837
CDTgdt	0.921	0.971	0.833	0.741	0.935	0.461	0.875	0.952	0.629
CDMgdt	**0.983**	**0.994**	**0.992**	0.643	0.968	0.372	0.881	0.968	0.657
CDAgdt	0.969	**0.990**	**0.991**	0.860	**0.976**	0.748	0.935	0.971	0.690
SMOC + OV	0.976	**0.989**	**0.991**	0.852	0.942	0.710	**0.976**	**0.976**	**0.976**
SCCC + OV	**0.982**	**0.990**	**0.991**	0.854	0.961	0.736	**0.974**	**0.975**	**0.970**
LMI + OV	0.969	**0.989**	**0.991**	**0.931**	**0.975**	0.855	**0.976**	**0.975**	**0.969**

**Supplementary Fig. S4:**
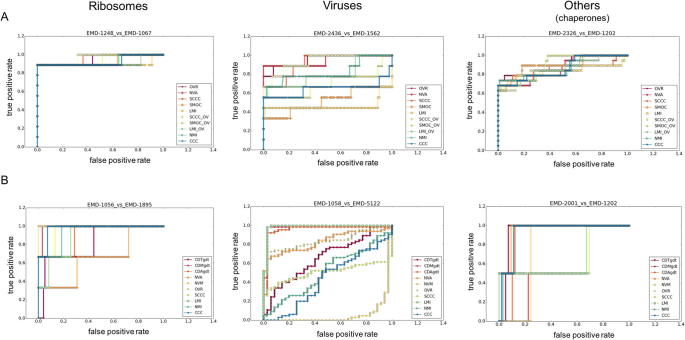
Performance of density and surface-based scores. ROC curves for (A) density based and (B) surface based scores, plotted for examples from each category: ribosomes, viruses, ‘others’.

The normalization of CD scores based on GDT-like weights improved their AUC values and precision, especially in the case of ribosomal and ‘others’ (Table 2, Figs. 4 & S3). The normalized CDAgdt and CDMgdt scores had the best AUC and accuracies for ribosomes, when compared to all the other scores (Table 2). Ribosomes have unique and discernible surface features when compared to viral maps and those belonging to the category ‘others’. Fig. S4B gives examples of cases from each category where the AUC of CD scores were comparable or better than other scores with good performance in each category. For example, in the case of viral map fit EMD-1058 (18 Å map of echovirus type 12 bound to a protein decay-accelerating factor (CD 55)) vs EMD-5122 (10 Å map of human poliovirus 1 RNA-releasing intermediate), the performance of CDMgdt score better than other scores except LMI, which also had a similar ROC curve. Though the echovirus is packaged, the maps have similar exposed surface features represented by viral proteins VP1 and VP3 (Fig.1B and S4B). The CDMgdt score was effective in distinguishing alignments base on these exposed surface features (Fig.1B).

**Supplementary Fig. S5:**
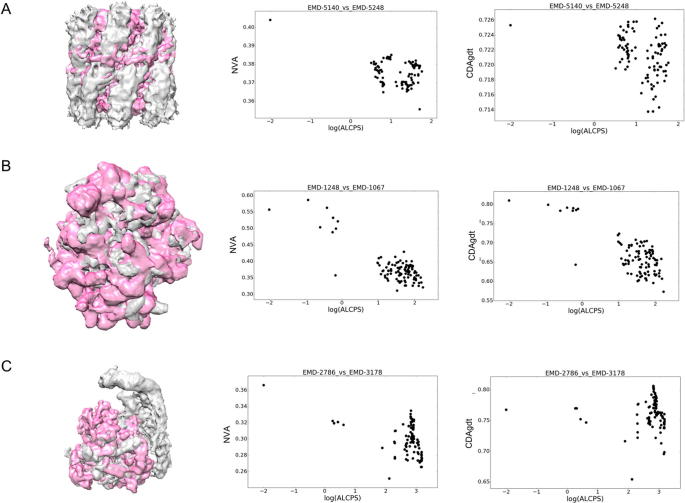
Examples of map alignments involving significant conformational differences. The plots show the scores of 100 fits generated by GMfit, the dot in the top left-hand corner which indicates the reference fit (the reference alignment is assigned a minimum log_10_(ALCPS) value of −2.0 for the purpose of plotting). ALCPS measures the distance of a fit from the reference alignment, with lower values indicating better fits. See the Methods for an explanation of the different scores shown here.

In the selected dataset, there are many cases in the viral and chaperone map pairs where both outer and inner surfaces have significant differences due to nucleic acid packaging (e.g. EMD-2436 vs EMD-1562, EMD-5466 vs EMD-2397, EMD-1058 vs EMD-5122 etc), substrate binding (e.g. EMD-2325 vs EMD-2326) and conformational changes (e.g. EMD-5140 vs EMD-5248). NVA, which is calculated by comparing gradient normals at all surface points, has generally higher AUC and precision for viral maps (Table 2, Fig. 4 & S3), comparable to the best density-based scores. As the score works based on density gradient vectors, it is less affected by the differences in the location of selected surface voxels in comparison to the CD scores. Fig. S5 gives a few examples where the maps being compared have significant conformational differences. Even in these difficult cases of alignment, the surface based score NVA, is useful in discriminating the correct alignments from the rest.

### 3.3 Local vs global density-based scores

Local cross correlation (SCCC) was introduced to avoid the influence of non-overlapping density in the calculations and quite a few developments that followed used this score (Roseman, 2000, Trabuco et al., 2008, Velazquez-Muriel et al., 2005). This is especially relevant in the case of subunit matching where the density of other components contribute to global score calculations. Local score calculations do not suffer from these limitations but they do not account for the extent of overlap. In other words, a small overlapping segment can have a better correlation score than a relatively larger overlap. An example is shown in Fig. S2, for the alignment of viral maps: Enterovirus 71 empty capsid (EMD-5466) and Enterovirus 71 in complex with a neutralizing antibody E18 (EMD-2397). Some of the incorrect fits with minimal overlap get higher SCCC scores compared to correct orientations (log_10_(ALCPS) < −1.0) with higher overlap. In terms of precision, SCCC was best for ribosomes and was among the top few scores for the group, ‘others’. (Table 2, Fig. S3).

### 3.4 Addition of overlap information to local scores

Generally, the fraction of overlap (OVR) score was good at discriminating between good and bad alignments across all structural categories, as judged by the AUC (Fig. 2), but had relatively lower precision (higher false positives) for the category ‘others’ including chaperones (Table 2). OVR score by itself is a good measure to discriminate correct alignments but is independent of voxel density values. Especially in case of subunit alignments or in the absence of significant surface features, one encounters solutions where most or all have large overlap with the target volume and hence OVR is less discriminatory in this context. An example from our dataset is the alignment of two ribosomal reconstructions: the partial yeast 48S preinitiation complex (EMD-2763) and E-*coli* 30S subunit in complex with the YjeQ biogenesis factor (EMD-1895). The reference alignment scored lower than the bad fits by OVR metric (Fig. S2). SCCC, however, picks the reference fit with the best score. Also, in theory, two different but similarly-sized sub-volumes will fit with the same overlap score at a specific region of the target map. Hence a combination of correlation score with the overlap information could be more suitable. We calculated combined scores after scaling OVR relative to other scores (e.g. SCCC) by applying scale and shift factors as:

$$\mathrm{Scale}\;\mathrm{factor}=\frac{\mathrm{median}\;\mathrm{absolute}\;d\mathrm{eviation}\;\mathrm{of}\;\mathrm{SCCC}}{\mathrm{median}\;\mathrm{absolute}\;\mathrm{deviation}\;\mathrm{of}\;\mathrm{OVR}}$$

Shiftfactor=(medianofSCCC)-(medianofOVR)

The OVR score was first scaled and then shifted by a shift factor.

OVRnorm=(OVR∗scalefactor)+shiftfactor

The combined score is the average of scaled and shifted OVR (*OVRnorm*) and SCCC/SMOC/LMI.

Inclusion of OVR information to the local scores (SCCC + OV, SMOC + OV and LMI + OV) improved the AUC significantly for all the three categories (Table 2, Fig. 4). These scores had comparable or better AUC and precision values than the best scores in each category (Table 2). LMI + OV (LMI_OV in the Fig. 4) had better precision than the other two correlation-based scores, especially in the case of viral maps. Fig. S4 gives examples of cases from each category with the performance of different scores highlighted by ROC curves.

**Fig. 5:**
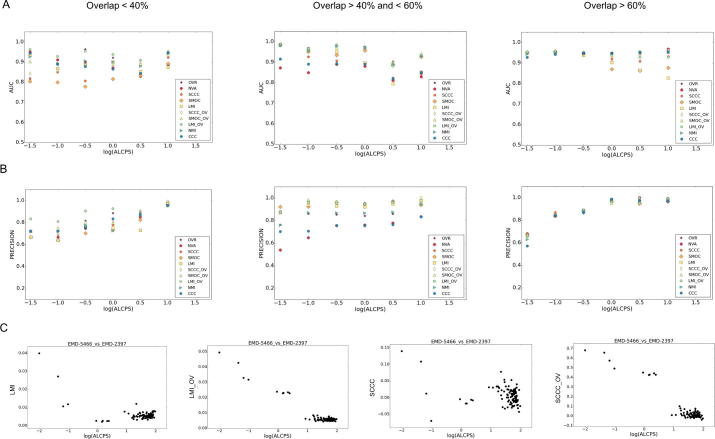
AUC and precision of scores vs deviation from the reference alignment. The figure shows (A) AUC values and (B) precision of density based scores and their combinations with OVR score for cases where the minimal percent of overlap (out of the two maps) in the reference alignment is <40%, between 40 and 60% and >60%. (C) Scores vs ALCPS (log_10_ scale) for an example: EMD-5466 vs EMD-2397, highlighting improvement in discriminating true and false alignments with the addition of overlap information. Fits with ‘correct’ orientations are below log_10_(ALCPS) −0.5. OVR: Overlap score, LMI: Local mutual information, NMI: Normalized mutual information, SCCC: Local cross correlation, SMOC: Local cross correlation about zero. The combined scores with OVR are indicated with the ‘_OV’ tag. CDT: Surface distance score on points selected based on a density threshold range, CDM: Surface distance score on points selected using mean filter (to identify more exposed regions), CDA: Surface distance score on all points at an iso-contour level, CDMgdt & CDAgdt scores are normalized variants of CDM & CDA (see Methods), NVM: Normal vector score on surface points identified by mean filter on binary mask, NVA: Normal vector score on all points at an iso-contour level.

The combined scores were better than most other scores when the maps overlap partially (Fig. 5). Overall, the LMI + OV score had the best AUC for cases where only part of the maps match (<60% overlap) (Fig.5A). In terms of precision, LMI + OV was also among the best scores (Fig.5B). As mentioned above, global scores are as effective when major portions of the maps overlap (Fig.5A&B).

### 3.5 Performance at different resolution ranges

**Fig. 6:**
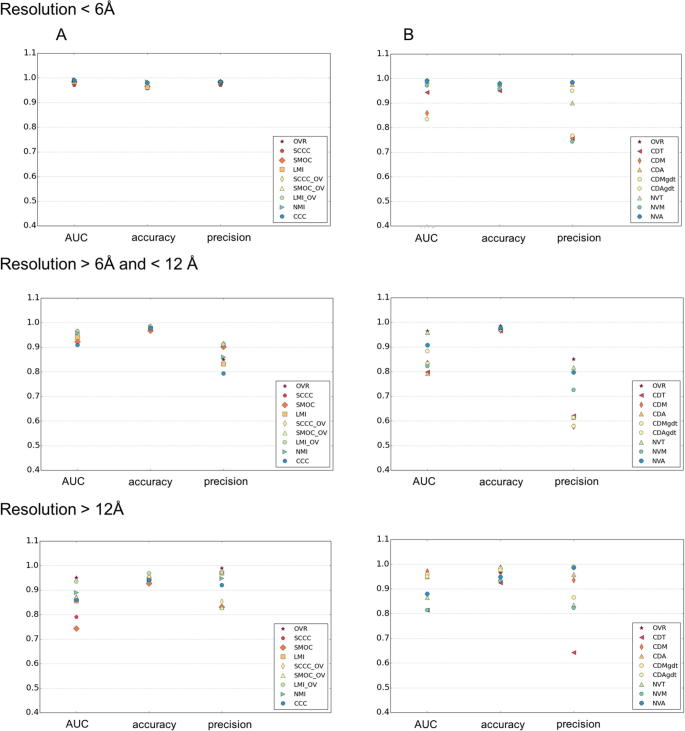
Performance of density and surface based scores at different resolution ranges. The AUC, accuracy and precision of density (left) and surface based (right) scores are plotted for maps of resolutions (lower of the two maps) better than 6 Å, 6–12 Å and worse than 12 Å (5, 11 and 8 cases respectively). The AUC and precision values are calculated at log_10_(ALCPS) thresholds selected for each category (ribosomes: 0.82, viruses: −0.5, chaperones: −0.4). OVR: Overlap score, LMI: Local mutual information, NMI: Normalized mutual information, SCCC: Local cross correlation, SMOC: Local cross correlation about zero. The combined scores with OVR are indicated with the ‘_OV’ tag. CDT: Surface distance score on points selected based on a density threshold range, CDM: Surface distance score on points selected using mean filter (to identify more exposed regions), CDA: Surface distance score on all points at an iso-contour level, CDMgdt & CDAgdt scores are normalized variants of CDM & CDA (see Methods), NVM: Normal vector score on surface points identified by mean filter on binary mask, NVA: Normal vector score on all points at an iso-contour level.

Analysis of different scores for their performance with maps at different resolution ranges (Fig. 6), shows that at resolutions better than 6 Å, all the density-based scores and OVR has similar AUC and precision (Fig. 6). At intermediate and low resolutions, the scores involving combination of SCCC, SMOC or LMI and OVR scores, had better AUC and precision than other scores. LMI + OV score (LMI_OV in the figure) was slightly better at low resolutions compared to the combined scores involving SCCC or SMOC. This is largely due to the fact that the MI scores use a coarser or binned representation of density, which is useful at these resolutions. Among the surface-based scores, NVA was better overall at both high (better than 6 Å) and intermediate resolutions (6–12 Å). CDAgdt score was better discriminatory at resolutions worse than 12 Å, apart from the combined scores.

### 3.6 Performance when fitting atomic components to maps representing a larger complex

**Fig. 7:**
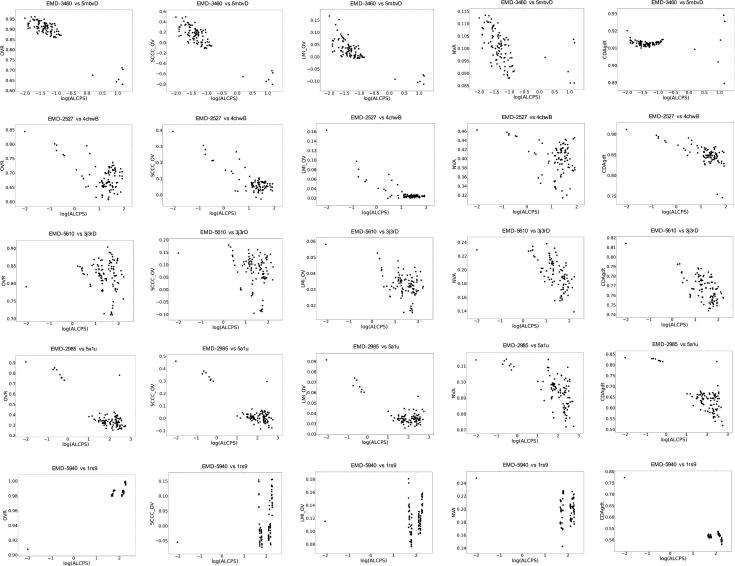
Scores vs ALCPS (log_10_ scale), for cases of subunit model fits in maps. In the plots, each point represents one of the 100 fits generated by GMfit, except for the dot in the top left-hand corner which indicates the reference fit (The reference alignment is assigned a minimum log_10_(ALCPS) value of −2.0 for the purpose of plotting). ALCPS measures the distance of a fit from the reference alignment, with lower values indicating better fits. See the Methods for an explanation of the different scores shown here.

We also tested the performance of different scores for discriminating the reference and near-optimal fits from incorrect fits when fitting a component of the complex represented by the map. We selected 5 cases spanning different resolutions (Table S1). The fitted model associated with the map (in PDB) was considered as the reference fit and the component was fitted in the map using GMfit. While assessing the solutions from GMfit, we observed trends similar to that of the whole-map alignments where the density based scores were better at higher resolutions and the surface-based scores were more discriminatory at low resolutions (Fig. 7). Only in the case EMD-5940 vs PDB 1rs9, where the crystal structure of nitric oxide synthase heme domains (dimer) were fitted into the low resolution (23 Å) map of calmodulin bound dimeric nitric oxide synthase, the density based scores failed and only the surface scores identified the correct fit (Yokom et al., 2014) (Fig. 7). The density-based and combined scores were less discriminatory at this resolution. LMI + OV was better across resolutions when compared to SCCC + OV (e.g. EMD-5610 vs 4chwB in Fig. 7). As mentioned above, a simple OVR score is not effective as a general metric for cases of partial fits (e.g. EMD-5940 vs 1rs9, EMD-5610 vs 3j3rD).

### 3.7 Computational speed

All the scoring functions tested in this study (new scores and the improvements from our previous studies (Farabella et al., 2015, Vasishtan and Topf, 2011)) can be used to rank a set of alignments generated by any density fitting method, and are suitable for comparing either two volumes or an atomic model and a volume at any resolution. They are all implemented in TEMPy (http://tempy.ismb.lon.ac.uk/). When applied to maps of size 300 × 300 × 300 using a single processor, the calculation takes: ∼0.5 s for LMI + OV (combined score) and CD (surface-based), ∼0.9 s for NVA (surface-based), and ∼0.05 for SCCC + OV (local density-based). The longer run-time of LMI + OV score can be attributed partly to the time for generation of binned density maps and calculation of frequencies, and also to the current Pythonic implementation. We plan to re-implement this metric in C, which will improve the speed of calculations.

## 4 Summary and recommendations

As part of a volume-matching pipeline that we have been developing for 3D-EM data, we have tested various approaches to score alignments obtained from volume matching programs. We demonstrate that the performance of the different scoring functions varies depending on the shape and density composition of the assemblies represented by the map, the resolution and the extent of similarity or overlap.

**Fig. 8:**
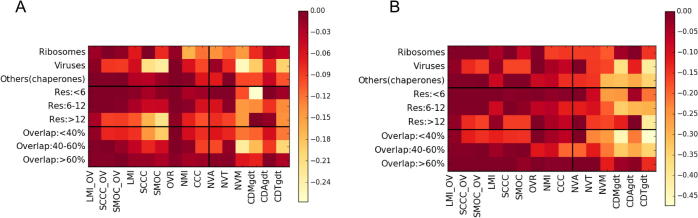
Summary of performance of scores in alignment groups categorized based on map-types, resolutions and fraction of overlap (minimum overlap fraction with respect to the two map sizes). For each group (row in the matrix-plot), differences from the maximum (A) AUC and (B) precision, are plotted. The AUC and precision values are calculated at log_10_(ALCPS) thresholds selected for each category (ribosomes: 0.82, viruses: −0.5, ‘others’: −0.4). Resolution (Res) is in Å. OVR: Overlap score, LMI: Local mutual information, NMI: Normalized mutual information, SCCC: Local cross correlation, SMOC: Local cross correlation about zero. The combined scores with OVR are indicated with the ‘_OV’ tag. CDT: Surface distance score on points selected based on a density threshold range, CDM: Surface distance score on points selected using mean filter (to identify more exposed regions), CDA: Surface distance score on all points at an iso-contour level, CDMgdt & CDAgdt scores are normalized variants of CDM & CDA (see Methods), NVM: Normal vector score on surface points identified by mean filter on binary mask, NVA: Normal vector score on all points at an iso-contour level.

Overall, our results (summarised in Fig. 8) show that combined scores are more effective as a general measure than the standard CCC, which is not the best discriminator across all resolutions or for partial overlaps. A combined score involving local mutual information and fraction of overlap (LMI_OV in the figure) is the best performing score in terms of AUC and precision across resolutions, map types and extents of overlap. *We therefore recommend LMI_OV as the preferred scoring function for general use, while other scores may be useful for studies focused on particular cases*.

Generally, *density-based scoring functions* are influenced by the size and shape of the density distributions being compared while surface-based scoring functions are affected mainly by the extent of surface features and selection of contour. When comparing maps that typically have partial overlaps and significant compositional differences (e.g. viral maps), mutual information-based scores (NMI/LMI) are more discriminatory than cross-correlation-based scores (CCC/SCCC/SMOC). However, for ribosomal maps and ‘others’, the local SCCC score has better precision, at high-to-intermediate resolutions in cases where the maps overlap to a large extent.

*Surface-based scoring functions* can also be useful at intermediate-to-low resolutions. We find that surface detection by selection of all points on the iso-contour (surface definition A) is generally more effective for such scores. Among these, the normal vector score (NVA) calculated based on surface density gradients, is more precise at different resolution ranges (mainly high-to-intermediate) especially when there are significant compositional and conformational differences (e.g. in the case of viruses and chaperones). The Chamfer distance (CDAgdt), which is based on distance between surface points, is better at low resolutions, where density variation is less informative. In the future, we plan to develop approaches to detect local surface matches (partial overlaps) and test the performance of the surface-based scoring functions on local surface regions.

In summary, we have analysed a wide variety of scoring functions for comparing EM maps taken from a range of structural classes with different shape, size and resolution. We also provided combined metrics that include the information on the extent of overlap between volumes. Based on our results, we recommend the combined score LMI_OV as having the widest applicability. This score is likely to be useful for comparing large datasets of density maps and models and for integrative structure modelling based on data at different resolutions.
