# Supplementary material for: Improved metrics for comparing structures of macromolecular assemblies determined by 3D electron-microscopy
Source: J Struct Biol. 2017 Jul;199(1):12–26. doi: 10.1016/j.jsb.2017.05.007 (PMC5479444; doi:10.1016/j.jsb.2017.05.007)
Supplement: Supplementary Table S1 [file mmc6.docx]

| **EMD1** | **PDB,chain** | **Resolution** | **gmm1** | **gmm2** | **Fractional**  **Overlap**  **(m1,m2)** |
| --- | --- | --- | --- | --- | --- |
| 3460 | 5mbvD | 3.8 | 95 | 38 | 0.11, 0.96 |
| 2527 | 4chwB | 7.0 | 24 | 6 | 0.22, 0.85 |
| 5610 | 3j3rD | 10.0 | 18 | 4 | 0.11, 0.79 |
| 2985 | 5a1u | 13.0 | 28 | 6 | 0.16, 0.91 |
| 5940 | 1rs9* | 23.0 | 5 | 4 | 0.10,0.91 |

**Table S1**. Dataset used for evaluating scoring functions for subunit model search in a map. The EMDB IDs of the volumes aligned, the chains from the fitted model corresponding to the subunit used as search object, the map resolution and the number of gaussian density functions used for each (gmm1 and gmm2), are given. The fraction of overlapping region from the reference alignment with respect to the size of the map and model, is given in the last column. The reference fit of the subunit model in this case is generated manually to match with the fit proposed by the authors (Yokom et al. 2014).
